# Import of proteins into peroxisomes: piggybacking to a new home away from home

**DOI:** 10.1098/rsob.150148

**Published:** 2015-11-18

**Authors:** Sven Thoms

**Affiliations:** Department of Child and Adolescent Medicine, University Medical Center, University of Göttingen, Robert-Koch-Str. 40, 37075 Göttingen, Germany

**Keywords:** peroxisome, protein import, cellular organelles, piggyback import, Pex5, PTS1

## Abstract

Peroxisomes are capable of importing folded and oligomeric proteins. However, it is a matter of dispute whether oligomer import by peroxisomes is the exception or the rule. Here, I argue for a clear distinction between homo-oligomeric proteins that are essentially peroxisomal, and dually localized hetero-oligomers that access the peroxisome by piggyback import, localizing there in limited number, whereas the majority remain in the cytosol. Homo-oligomeric proteins comprise the majority of all peroxisomal matrix proteins. There is evidence that binding by Pex5 in the cytosol can regulate their oligomerization state before import. The hetero-oligomer group is made up of superoxide dismutase and lactate dehydrogenase. These proteins have evolved mechanisms that render import inefficient and retain the majority of proteins in the cytosol.

Peroxisomes are curious organelles. Among their at times bewildering features is their apparent and often quoted ability to import folded and even oligomeric proteins [1,2]. The capability of peroxisomes to import oligomers is tied to a related phenomenon: not all subunits of the oligomer require a peroxisomal targeting signal (PTS). It is sufficient for the co-import of other subunits of a complex when one subunit carries a PTS [3,4]. Such co-import is also termed piggyback import.

One of the experiments showing piggyback oligomer import was conducted with transient overexpression of the peroxisomal oilseed isocitratelyase (IL) or the bacterial reporter protein chloramphenicol acetyltransferase (CAT) in cell culture [3,5]. IL is a homo-tetramer, CAT a homo-trimer and both carried a peroxisomal targeting signal type 1 (PTS1): IL is a natural protein of the plant peroxisome (glyoxysome), and in the case of CAT, a PTS1 was added at the C-terminus. The authors noted that IL and CAT could still be imported into the peroxisome when the PTS1 was deleted and when at the same time wild-type IL or CAT-PTS1, respectively, were co-expressed [5]. This experiment and many others showed that PTS1-less proteins can be piggybacked into peroxisomes when co-expressed in the same cell with interacting subunits carrying a PTS [1,2]. Taken together, these studies showed that proteins pass the membrane into peroxisomes as oligomers.

Structural analysis of the yeast peroxisomal hydrolase Lpx1 contributed to this concept [6,7]. Lpx1 is a homodimer; the two subunits embrace each other by the C-terminal alpha-helix that, at its very terminus, also contains the PTS1 [7]. When the embracing helix is removed, the interaction of the Lpx1 protomers is not interrupted, showing that dimerization is very robust and probably occurs once synthesis of the protomers is completed. Moreover, the dimer was efficiently imported into peroxisomes [7]. Dimerization must precede import, unless specific chaperones keep the protomers in a monomeric state.

Analysis of piggyback import of candidate cargo can be accomplished by a special kind of two-hybrid assay that is based on the expression of two fusion proteins: cyan fluorescent protein (CFP) fused to the N-terminus of one protein and yellow fluorescent protein (YFP) fused to the N-terminus of the other, PTS1-less protein [7]. In the case of piggyback transport, the PTS1-less cargo is only imported into peroxisomes when both constructs are co-expressed.

The peroxisomes' unusual ability to import oligomers is well known. However, it is presently a matter of debate as to whether most of the proteins found in peroxisomes are mainly translocated as oligomers or as monomers. A recent study added an important piece to this puzzle [8]: a thorough *in vitro* analysis of the targeting of acyl-CoA oxidase 1 (ACOX1) and urate oxidase (UOX) suggested that both proteins enter the peroxisome preferably as monomers, and binding of the cytosolic import receptor protein Pex5 can impede oligomerization [8]. At least in these two cases, the monomer is preferred, even though those proteins can be imported as oligomers.

Peroxisomal piggyback import of the lactate dehydrogenase (LDH) subunit A (or M for muscle) together with subunit B (or H for heart) is a different case, however. LDH has for several decades been implicated in peroxisome metabolism [9,10], but in the absence of a PTS on LDH, it has been difficult to understand the mechanism of translocation [11]. Recently, we conducted a genome-wide search for mammalian proteins which, similar to fungal proteins [12], can enter the peroxisome after functional translational readthrough of their mRNA. To find such proteins, we combined a novel predictor for stop codon readthrough (readthrough propensity, RTP) with a predictor of PTS1 targeting signals that would be hidden in-frame in the 3′UTR. The B subunit of LDH showed the highest combined RTP and hidden-PTS1 score [10]. Significantly, translational readthrough of about 2% together with the hidden targeting signal was shown to be responsible for the LDHB import into peroxisomes. When the hidden PTS in LDHB is deleted, the protein is withheld in the cytosol. Interestingly, targeting efficiency could be modulated by mutating the stop codon: when the stop codon was changed for a sense codon, the amount of LDHB in the peroxisome increased by nearly two orders of magnitude. On the other hand, when the leaky (readthrough-prone) stop codon context was exchanged for a stop codon with a lower RTP, the amount of LDHB in the peroxisome was reduced [10]. The peroxisomal content of LDHB could be increased by readthrough-inducing drugs which strongly suggest that peroxisomal localization of LDHB is, indeed, dependent on translational readthrough and the hidden targeting signal [10]. The readthrough form of LDHB is termed LDHBx (x stands for e*x*tended).

In these experiments, the large fraction of LDHB that was not associated with cellular organelles had to be removed, because 2% translational readthrough renders LDHBx a peroxisomal protein. The other 98% remained in the cytosol, thus masking the peroxisomal fraction of LDHBx [10]. When analysing peroxisomal import of a protein that is only an inefficient import substrate, the cytosolic pool masks the peroxisomal pool, and thus assessment of the actually imported protein becomes difficult [8].

LDH is a tetrameric protein that can be built by any combination of the two subunits A and B. With LDHBx, four of the five possible isoforms (all except A_4_) can directly be imported into the peroxisome by piggyback transport. Using the two-hybrid assay described above, it could be shown that translational readthrough and the hidden targeting signal are responsible for co-import into peroxisomes [10].

Since the mid-1990s, many studies have analysed the peroxisomes' potential of piggyback import. But until today, there are only two examples of natural piggyback import into peroxisomes. One concerns the co-import of LDHA and LDHB together with LDHBx, as detailed above. The only other reported natural piggyback substrate is superoxide dismutase (SOD1), which acquires entry into the peroxisome by interaction with the PTS1-bearing copper chaperone of SOD1 (CCS) [13].

There are at least two marked differences between homo-oligomer import and the two cases of hetero-oligomer import (LDHBx-LDHA/B and CCS-SOD1). One of the differences concerns the binding mode and function of Pex5 in regulation of oligomerization, and the other the major localization of the proteins. These will be discussed separately below.

In the case of homo-oligomer import, the co-imported subunits also contain PTS. It is still not clear whether more than one PTS are actually used, i.e. whether they are also bound to Pex5 during import. However, if the Pex5 binding site does not overlap with the dimerization surface as is the case, e.g. in Lpx1, both subunits would be able to bind Pex5 at the same time, and Pex5 binding would not inhibit oligomerization (figure 1*a*). If the PTS1 is in the vicinity of the dimerization surface, the binding of Pex5 could prevent dimerization (figure 1*b*), and thus dimer import could not take place. The data presented in a recent study suggest that in ACOX1 and UOX import, Pex5 partially hinders dimerization [8]. This is in agreement with a structural analysis of these proteins. The C-termini in the ACOX1 dimer are close to the protomer interface (figure 2*a*), so it is possible that Pex5 binding renders the dimerization less effective. Furthermore, the termini in the UOX tetramer are close to the protomer interaction face, and it is therefore conceivable that Pex5 interferes with dimerization (figure 2*b*).

**Figure 1.**
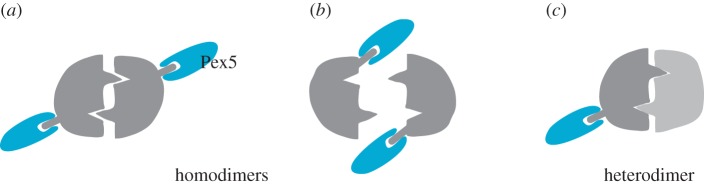
Modes of dimerization with respect to Pex5 binding. (*a*) Homo-dimerization of a PTS1 protein with distal PTS1 termini. Up to two molecules of Pex5 can bind to the dimer. The relative sequence of dimerization is open: Pex5 can also bind to the monomer and monomer import can precede dimerization. Peroxisomes have the ability to import dimers. During import, one or two molecules of Pex5 could be bound to the dimer. (*b*) When the PTS1 is close to the dimerization interface, it is possible that Pex5 binding interferes with dimerization. PTS1 is at the C-terminus and is usually flexible, protruding from the protomer. This situation must therefore be finely balanced, because when the PTS1 is too close to the interaction surface, it would interfere with dimerization—and the protein would not be a dimer at all. Note that the PTS1 is not cleavable. (*c*) In hetero-oligomer import, only one of the subunits contains a PTS. Import of the other subunit(s) is strictly dependent on the PTS1-bearing subunit. The two known cases of hetero-oligomer import concern protein with dual localization, the major localization being in the cytosol, and only a small portion piggybacks into the peroxisome, either by interaction with a chaperone that is expressed at low level, or by functional translational readthrough of one of the subunits. The mode depicted here is also applicable for hetero-oligomers with more than two subunits.

**Figure 2.**
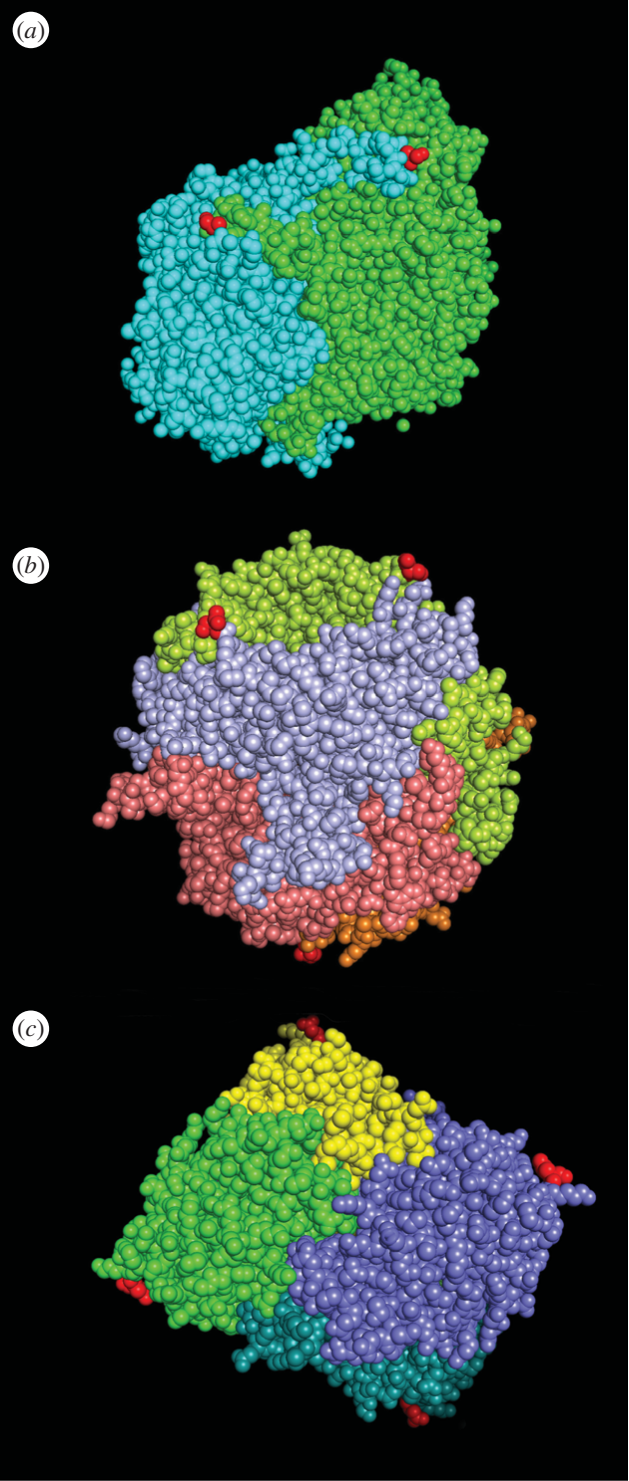
Different modes of oligomerization of peroxisomal proteins. C-termini of oligomeric peroxisomal matrix proteins. The last resolved amino acid at the C-terminus is drawn in red to indicate the (beginning of) the PTS1. (*a*) ACOX1 dimer. The last six amino acids are not resolved, probably because the termini maintain the flexibility that is required for Pex5 binding. The structure was drawn according to PDB 1IS2. (*b*) UOX tetramer. As in (*a*), the PTS1-bearing termini are close to the protomer interaction face, which is in agreement with a model in which subunits are imported as monomers and oligomerize in the peroxisome. The structure was drawn according to PDB 4OQC. (*c*) LDHB tetramer. The termini are at maximal distance to the protomer interaction face. It is unlikely that Pex5 binding to LDHBx (readthrough-extended LDHB) interferes with oligomerization. The cellular concentration of LDHBx is about 2% as defined by the degree of functional translational readthrough. In the case of this peroxisomal form of LDH, readthrough rather than Pex5 binding determines the dual localization equilibrium. The structure was created from PDB 1I0Z. The all-LDHB homo-tetramer (LDH-1) is shown to illustrate the position of the PTS1. In the cell, however, owing to the low concentration of LDHBx, peroxisomal LDH tetramers will contain only one LDHBx subunit in combination with LDHB and/or LDHA. All structures were generated using Pymol.

The substrate structure must be finely balanced to allow regulation of the oligomerization state by Pex5. When not bound to Pex5, the terminus should not inhibit protomer interactions, because, otherwise, the protein would always remain a monomer. On the other hand, the terminus with the Pex5 binding site must be close enough to the interaction site, so that Pex5 binding can block oligomerization, the cargo can remain monomeric before import, and oligomerization can occur only upon entry into the peroxisome. Only when the terminus is close—but not too close—to the protomer interface can Pex5 binding control the oligomerization status for effective import.

For hetero-oligomer import (figure 1*c*), it is not possible that dimerization occurs after import simply, because otherwise the PTS-less subunit would not be imported. It is also not possible for Pex5 binding to inhibit oligomerization, as is the case in homo-oligomers, because there would be no import of the PTS1-less subunit(s) of the complex. Furthermore, several Pex5 molecules cannot bind to an oligomer, because there is only one PTS per oligomer. Hetero-oligomers are thus strictly dependent on piggybacking. If oligomer import is generally less efficient than monomer import, the hetero-oligomer must pay the price, simply because there is no other way into the peroxisome.

The second important difference between homo-oligomer and hetero-oligomer import concerns the major localization of the oligomer. In both known cases of hetero-oligomer import, SOD1 and LDH, the major localization of these proteins is the cytosol. By interacting with CCS and LDHBx, SOD1 and the holoenzyme LDH have found a way of being imported into the peroxisome, yet still the bulk of the protein remains in the cytosol. In this sense, the import is already inherently ineffective, because only a small fraction of the cytosolic proteins are imported into the peroxisome. Import depends on the interaction of a cargo (SOD1 or LDHA/LDHB) with a carrier (CCS or LDHBx), and the resulting dual localization is controlled by the amount of available carrier. The relative concentration of CCS in the cytosol is lower than that of SOD1 [13], and the ratio of LDHBx (with the PTS1) to conventional LDHB is roughly 1 : 50 [10].

Hetero-oligomer import can be doubly inefficient: kinetically inefficient, because the peroxisome might prefer monomers over oligomers, and inefficient in amount, with the import of only 1/50 or 1/100 of a cytosolic protein into the peroxisome. Describing hetero-oligomer import as inefficient, however, might lead to false conclusions, because the peroxisome volume in an average cell is in the range of 1/100 of total cell volume. In conclusion, by importing 1/100 of the cytosolic SOD1 or LDH into the peroxisome, the concentrations of these proteins in the cytosol and the peroxisome are approximately equal. In the case of LDH, this is what is needed to maintain a cytosol–peroxisome shuttle for redox equivalents [10].

The concepts ‘oligomer import’ or ‘piggy-backing’ subsume two cases that need to be distinguished from each other: homo-oligomers on one side, and piggyback oligomers with dual localization on the other. Homo-oligomers can evolve their mode of receptor binding. If monomer import is generally more efficient than oligomer import, Pex5 binding could control the substrates' oligomerization state. Pex5 may therefore fulfil a double function as import receptor and regulator of oligomerization, thus preventing premature cargo oligomerization. Hetero-oligomers, which contain only one PTS, do not have that choice. They may profit from kinetically inefficient and stoichiometrically incomplete import to achieve their tightly controlled dual localization equilibrium.
